# Bioinformatics-Based Analysis of Ferroptosis-Related Biomarkers and the Prediction of Drugs Affecting the Adipogenic Differentiation of MSCs

**DOI:** 10.3390/biomedicines13040940

**Published:** 2025-04-11

**Authors:** Jiahao Jin, Zihao Yuan, Xinglang Wang, Quanfeng Li, Yunhui Zhang, Yibin Zhang, Pengfei Ji, Yanfeng Wu, Peng Wang, Wenjie Liu

**Affiliations:** 1 Department of Orthopedics, The Eighth Affiliated Hospital, Sun Yat-sen University, Shenzhen 518033, China; jinjh8@mail2.sysu.edu.cn (J.J.); yuanzh33@mail2.sysu.edu.cn (Z.Y.); wangxlstar@163.com (X.W.); liqf33@mail2.sysu.edu.cn (Q.L.); zhangyh289@mail2.sysu.edu.cn (Y.Z.); zhangyb78@mail2.sysu.edu.cn (Y.Z.); jipengf@mail2.sysu.edu.cn (P.J.); 2 Guangdong Provincial Clinical Research Center for Orthopedic Diseases, The Eighth Affiliated Hospital, Sun Yat-sen University, Shenzhen 518033, China; 3 Center for Biotherapy, The Eighth Affiliated Hospital, Sun Yat-sen University, Shenzhen 518033, China; wuyf@mail.sysu.edu.cn

**Keywords:** mesenchymal stem cells, osteoporosis, ferroptosis, adipogenic differentiation, molecular docking

## Abstract

**Background**: The imbalance between the osteogenic and adipogenic differentiation of mesenchymal stem cells (MSCs) is a key factor in the progression of osteoporosis; therefore, it is crucial to study the regulatory mechanisms that maintain this balance. Ferroptosis is a form of regulated cell death caused by the accumulation of lipid peroxides and is closely associated with various diseases. Changes in intracellular oxidative stress levels can affect the lineage allocation of MSCs. However, it remains unclear whether the disruption of intracellular oxidative stress levels caused by ferroptosis can influence the osteogenic–adipogenic differentiation balance of MSCs, and the mechanism underlying this influence in osteoporosis has not been fully elucidated. This study is the first to demonstrate through in vitro cell experiments that inhibiting ferroptosis can decrease the adipogenic differentiation of MSCs. **Methods and Results:** Through bioinformatics analysis, differentially expressed genes (DEGs) associated with the adipogenic differentiation of MSCs were identified from the GEO database. We then intersected these differentially expressed genes with a ferroptosis-related gene dataset and identified 118 ferroptosis-related differentially expressed genes (FRDEGs). Additionally, we explored the functional roles of FRDEGs through GO and KEGG analyses and found that these genes significantly impacted intracellular oxidative stress. Furthermore, we identified 10 key FRDEGs via protein-protein interaction (PPI) analysis. The diagnostic performance of these genes was evaluated by plotting receiver operating characteristic (ROC) curves, and the reliability of the diagmodel was validated using data from osteoporosis patients. We then constructed a mouse osteoporosis model and validated the mRNA expression levels of key FRDEGs via qRT-PCR, which revealed significant differences in expression in the osteoporosis group. Finally, molecular docking technology was used to identify two small molecules from the DrugBank database that are able to negatively regulate MSC adipogenic differentiation by inhibiting ferroptosis. **Conclusions:** The identified FRDEGs and small molecules offer novel diagnostic markers and therapeutic candidates for osteoporosis.

## 1. Introduction

Mesenchymal stem cells (MSCs) are pluripotent stem cells with multilineage differentiation potential that can be induced to differentiate into various mesodermal cell types, such as adipocytes and osteoblasts [1,2]. Typically, the balance between the osteogenic and adipogenic differentiation of MSCs is strictly regulated in both the temporal and spatial dimensions to ensure skeletal health. The disruption of this balance may result in the development of osteoporosis (OP) [3,4]. The theory of bone–fat imbalance suggests that MSC differentiation imbalance is influenced by various factors, such as MSC senescence and decreased MSC activity, which promote adipogenic differentiation while inhibiting osteogenic differentiation. The dysregulation of signalling pathways such as the Wnt/β-catenin and PPARγ pathways, as well as changes in the levels of cytokines, hormones, and mechanical stress in the local microenvironment, can also affect the balance between osteogenesis and adipogenesis in MSCs [5,6,7]. Furthermore, studies have shown [8,9] that MSCs from osteoporosis patients tend to differentiate into adipocytes. In both postmenopausal women and ovariectomized animals, a decrease in bone mass is observed along with an increase in bone marrow fat, but estrogen supplementation leads to a significant decrease in marrow fat content and restoration of bone mass, alleviating osteoporosis [10,11]. As a result, interventions aimed at regulating MSC adipogenesis may emerge as novel approaches for treating OP.

Ferroptosis is an iron-dependent form of programmed cell death, primarily involving the antagonism between intracellular oxidative stress and antioxidant defense systems. When oxidative stress induced by iron accumulation exceeds the antioxidant buffering capacity provided by the cell’s defense systems, particularly with the loss of glutathione peroxidase 4 (GPX4) activity, lipid peroxides cannot be effectively cleared. This results in oxidative damage to the membrane phospholipid bilayer, ultimately leading to cell death [12]. Ferroptosis has been shown to play an important role in the onset and progression of many diseases, such as cancer, inflammation, and cardiovascular diseases [13,14,15]. In recent years, increasing research has confirmed that ferroptosis may be a new target for the prevention and treatment of osteoporosis and that inhibiting ferroptosis in osteoblasts or osteocytes can effectively reduce bone loss [16,17]. In addition, Cen Luo et al. [18] used ferroptosis inhibitors to prevent the production of reactive oxygen species (ROS) and lipid peroxides (LPOs) in osteoblasts, thereby activating downstream Wnt signaling, which also inhibited the progression of osteoporosis. However, the impact of ferroptosis on MSC adipogenic differentiation and its specific mechanisms in osteoporosis remain unclear.

In this study, we first demonstrated through in vitro cell experiments that inhibiting ferroptosis can reduce the adipogenic differentiation of MSCs. We then analyzed an MSC adipogenic differentiation-related dataset from the Gene Expression Omnibus (GEO) database, identified differentially expressed genes (DEGs), and intersected these DEGs with ferroptosis-related genes to identify ferroptosis-related differentially expressed genes (FRDEGs) that affect MSC adipogenic differentiation. Next, a protein-protein interaction (PPI) network was constructed to identify key FRDEGs. The diagnostic value and expression of the hub genes were validated using data from external osteoporosis datasets and a mouse osteoporosis model. Then, promising drugs targeting the ferroptosis-related hub genes were identified from the DSigDB database, and molecular docking technology was used to simulate the binding of the small molecules to the protein products of the hub genes. Finally, the effects of these drugs on MSC adipogenic differentiation were validated. Therefore, this study has the potential to provide new biomarkers for the early diagnosis of osteoporosis and offers theoretical support for the development of new drugs for osteoporosis.

## 2. Materials and Methods

### 2.1. Data Download

The datasets related to adipogenic differentiation (GSE59450) and osteoporosis (GSE35956) were obtained from the GEO database. Both datasets focus on human mesenchymal stem cells as the studied cell type. The GSE59450 dataset comprises three adipogenic induction groups and three control groups without treatment. The platform used was GPL14550 Agilent-028004 SurePrint G3 Human GE 8x60K Microarray (Probe Name Version). The GSE35956 dataset comprises five elderly osteoporosis patients and five middle-aged non-osteoporotic donors, utilized to assess the diagnostic value of the hub genes. The platform used was GPL570 [HG-U133_Plus_2] Affymetrix Human Genome U133 Plus 2.0 Array. Furthermore, ferroptosis-related genes were retrieved from the FerrDb database (http://zhounan.org/ferrdb/, accessed on 20 January 2025).

### 2.2. Identification of DEGs

Using R software (version 4.4.1) and the limma package, we performed boxplots and principal component analysis (PCA) on the dataset to verify the downloaded data and identify DEGs, applying the selection criteria of |log2(fold change)| > 1 and *p*-value < 0.05. A Venn diagram of ferroptosis-related genes and DEGs was generated using the online tool Venny 2.1.0 (https://bioinfogp.cnb.csic.es/tools/venny/index.html, accessed on 20 January 2025). Heatmaps and volcano plots were created using the R packages “heatmap” and “ggplot2”, respectively.

### 2.3. GO, KEGG, and GSEA Enrichment Analysis

The R package “clusterProfiler” was used to perform GO, KEGG, and GSEA enrichment analyses, with the “org.Rn.eg.db” package utilized for gene annotation. A *p*-value of <0.05 was considered statistically significant. Finally, the R packages “ggplot2” and “enrichplot” were used for result visualization.

### 2.4. PPI Network Construction and Hub Gene Identification

The STRING database (http://string-db.org, accessed on 20 January 2025) was used to analyze ferroptosis-related differentially expressed genes, with a composite score > 0.7 selected as the cutoff, and a PPI network was constructed. Protein–protein interaction results were retrieved and analyzed using Cytoscape software (v3.6.1), generating an interaction network. The cytoNCA and MCODE plugins were employed to calculate the connectivity degree between genes.

### 2.5. Correlation Analysis Among Hub Ferroptosis Genes

Correlation analysis on key ferroptosis genes was performed using the “corrplot” package in R, and the correlation coefficients between genes were calculated. The “circlize” package in R was employed to create a chord diagram for correlations, and the “ggplot2” package was utilized to generate a correlation heatmap.

### 2.6. Diagnostic Value Analysis of Hub Ferroptosis Genes

To validate the potential diagnostic value of the ten hub genes, the osteoporosis dataset GSE35956 was analyzed. The pROC package was used to generate receiver operating characteristic (ROC) curves to assess the diagnostic performance of the hub genes. The area under the ROC curve (AUC) was calculated, with values ranging from 0 to 1; a higher AUC indicates better predictive performance [19]. Additionally, a nomogram model was constructed using the “rms” package, based on selected variables. The accuracy of the model was tested using calibration curves and ROC curves.

### 2.7. OVX Model Construction

OVX mice were generated by performing bilateral ovariectomy or sham surgery on 2-month-old female wild-type (WT) C57BL/6 mice. Following a 2-month recovery period after ovariectomy, the mice were euthanized and subjected to Micro-CT analysis.

### 2.8. Micro-CT Analysis

Micro-CT analysis was conducted using the Inveon MM system (Siemens, Knoxville, TN, USA) to assess bone structure. The images were captured through 360 rotational steps with a pixel size of 8.82 μm, a voltage of 80 kV, a current of 500 μA, and an exposure time of 1500 ms. Key parameters, including the bone volume/total volume (BV/TV) ratio, trabecular thickness (Tb. Th), trabecular number (Tb. N), cortical thickness (Ct. Th), and trabecular separation (Tb. Sp), were calculated using Siemens Inveon Research Workplace software (version 4.0).

### 2.9. MSC Isolation and Culture

Mouse mesenchymal stem cells were isolated from the femur and tibia. The MSCs were then isolated and cultured according to our previous study [20]. To induce osteogenesis, MSCs were cultured in osteogenic medium containing 10% FBS DMEM, 100 IU/mL penicillin, 100 IU/mL streptomycin, 0.1 μmol/L dexamethasone, 10 mmol/L β-glycerophosphate, and 50 μmol/L ascorbic acid. To induce adipogenesis, MSCs were cultured in adipogenic medium containing 10% FBS DMEM, 1 μM dexamethasone, 10 μg/mL insulin, 0.5 mM IBMX, 0.2 mM indomethacin, and 100 IU/mL penicillin–streptomycin. In our study, MSCs were cultured for a total of 14–21 days to complete differentiation assays, No signs of senescence were observed during this period. The osteogenic and adipogenic media were replaced every 3 days, with Oil Red O staining performed on day 7 and alkaline phosphatase staining on day 14.

### 2.10. Total RNA Extraction and qRT-PCR Analysis

Total RNA was isolated from MSCs using TRIzol reagent (Invitrogen, Waltham, MA, USA; #15596-026). Reverse transcription was performed using Evo M-MLV RT premix (AG, Changsha, China; #11706) according to the manufacturer’s protocol. RT-qPCR was performed using the SYBR Green Premix Pro Taq HS qPCR kit (AG, Changsha, China; #11718). Quantification was performed using the 2^−ΔΔCt^ method, and the data were normalized to GAPDH levels. The primer sequences for each gene are provided in Supplementary Table S1.

### 2.11. Alkaline Phosphatase (ALP) Assays

Prior to staining, MSCs were fixed at room temperature with 4% paraformaldehyde (PFA) for 30 min. MSCs were stained using the BCIP/NBT alkaline phosphatase kit (Beyotime, Shanghai, China; C3206). To assess ALP activity, MSCs were lysed in RIPA buffer (Sigma Aldrich, St. Louis, MO, USA; R0278). ALP activity kits (Nanjing Jiancheng Biotech, Nanjing, China; A059-2) were then used to assess ALP activity via quantification of spectrophotometric absorbance at 405 nm.

### 2.12. Oil Red O (ORO) Assays

After 7 days of adipogenic induction, the cells were washed with PBS and subsequently fixed with 4% paraformaldehyde for 30 min. ORO staining was performed using the Oil Red O staining kit (Beyotime, #C0157S). Briefly, the ORO staining solution was prepared by mixing the ORO solution and diluent in a 3:2 ratio, followed by filtration through a 0.45 μm filter. The cells were stained with the ORO solution at room temperature for 20 min. The cells were then washed three times with PBS, and images of the stained MSCs were captured under a microscope. For quantitative analysis, ORO was extracted by adding isopropanol, and the absorbance at 520 nm was quantified.

### 2.13. Drug Prediction and Molecular Docking

Potential targeted drugs for ferroptosis-related hub genes were predicted using the DSigDB database. The predicted compounds were ranked based on their composite scores, and the top 10 compounds were displayed in a bar chart. The molecular structures of the drugs were retrieved from the PubChem database and saved in SDF format. Gene IDs were retrieved from the UniProt database and imported into the PDB database to obtain the protein structures encoded by the genes. The receptor was selected and downloaded in PDB format. Water molecules and small molecules were removed using PyMOL software (version 3.1). Molecular docking between the compounds and gene-encoded proteins was conducted.

### 2.14. Cell Counting Kit-8(CCK-8) Assay

Cell viability was assessed according to the instructions provided, using the CCK-8 (Beyotime, Shanghai, China) assay kit. Briefly, MSCs were incubated overnight in a 96-well plate (5 × 10^4^ cells/well), followed by treatment with 10 μL of CCK-8 reagent at 37 °C for 2 h. The absorbance of MSCs at 450 nm was measured using a microplate reader (Thermo Fisher Scientific, Waltham, MA, USA).

### 2.15. Protein Extraction and Western Blotting

To extract proteins, cells were lysed in RIPA buffer (Beyotime Biotechnology, Shanghai, China; Cat. No. P0013) and centrifuged at 12,000 rpm at 4 °C for 30 min. To separate proteins, SDS-polyacrylamide gel electrophoresis (SDS-PAGE) was performed with a 5% stacking gel and a 10% separating gel, followed by transfer of proteins to a PVDF membrane (Merck Millipore, Burlington, MA, USA; Cat. No. IPVH00010). The membrane was blocked with Tween 20 (TBST) containing 5% skim milk for 1 h. After washing twice with PBS, the membrane was incubated with the primary antibody overnight at 4 °C. The membrane was incubated with the secondary antibody for 1 h at room temperature.

### 2.16. Statistical Analysis

Experimental data are expressed as the mean ± standard deviation. Differences between two independent groups were assessed using the unpaired Student’s *t*-test. Statistical analysis and graphing were performed using GraphPad Prism software (version 9.4.0), and differences were considered statistically significant when *p* < 0.05.

## 3. Results

### 3.1. Inhibition of Ferroptosis Reduced MSC Adipogenic Differentiation Induced by Iron Overload

**Figure 1:**
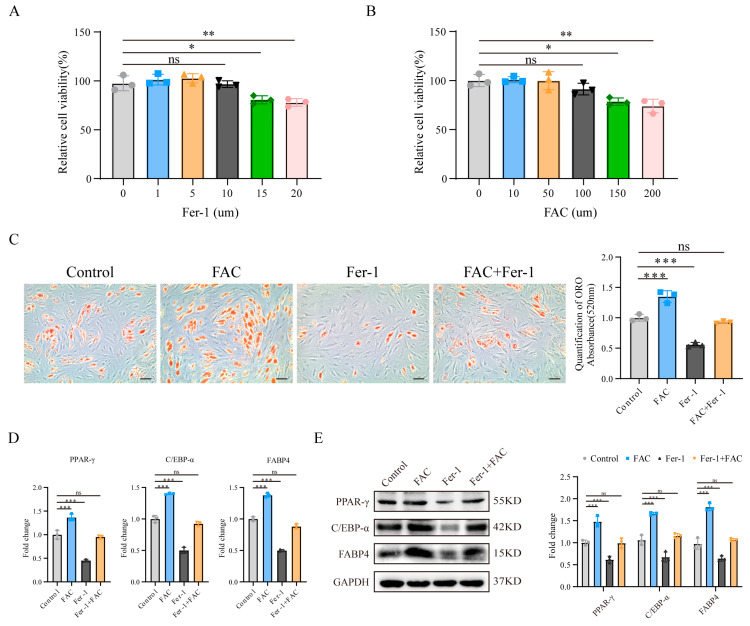
(**A**) Effects of exposure to different concentrations of Fer-1 (0, 1, 5, 10, 15, or 20 μM) on MSC viability. (**B**) Effects of exposure to different concentrations of ferric ammonium citrate (FAC) (0, 10, 50, 100, 150, or 200 μM) on MSC viability. (**C**) ORO staining and quantitative analysis of MSC adipogenic differentiation. The scale bar in the images represents 50 μm. (**D**) qRT-PCR and (**E**) Western blotting analysis of the expression of the adipogenic markers PPAR-γ, C/EBP-α, and FABP4 in MSCs. All the data are presented as the means ± SDs, with n = 3 per group, * *p* < 0.05, ** *p* < 0.01, and *** *p* < 0.001. ns, no significant difference. All experiments were independently repeated three times.

To explore the relationship between ferroptosis and the adipogenic differentiation of MSCs, we first established relevant cell models by using medium containing ferric ammonium citrate (FAC) and the ferroptosis inhibitor ferrostatin-1 (Fer-1) to generate a cellular environment that is conducive to ferroptosis or inhibits ferroptosis. FAC significantly increases the intracellular Fe^2+^ concentration and is an effective ferroptosis inducer [18], whereas Fer-1, a lipid peroxidation scavenger, is an effective ferroptosis inhibitor [21]. We evaluated the effects of FAC and Fer-1 on MSC viability at different concentrations using the CCK-8 assay. The results revealed that, within the concentration range of 0–10 μM, Fer-1 had no significant effect on MSC viability (Figure 1A); similarly, within the concentration range of 0–100 μM, FAC had no significant effect on MSC viability (Figure 1B). Therefore, in subsequent experiments, we treated MSCs with 10 μM Fer-1 and 100 μM FAC. When the cell density reached 60–80% confluence, we prepared osteogenic or adipogenic differentiation media containing appropriate concentrations of FAC/Fer-1 in advance and then added them to the cells to induce differentiation.

Next, we examined the adipogenic differentiation of MSCs. We used Oil Red O staining to evaluate the extent of adipogenic differentiation of MSCs from different groups after 7 days of induction. The results revealed that FAC significantly promoted MSC adipogenic differentiation, whereas Fer-1 not only significantly inhibited this effect but also reduced FAC-induced adipogenic differentiation (Figure 1C). In addition, we examined the expression levels of the adipogenesis-related markers PPAR-γ, CEBP-α, and FABP4. qRT-PCR and Western blotting revealed that FAC treatment increased the expression of these markers, while Fer-1 treatment significantly reduced their expression, and Fer-1 inhibited the FAC-induced upregulation of these markers (Figure 1D,E). These findings suggest that blocking ferroptosis in MSCs can effectively inhibit their adipogenic differentiation.

### 3.2. Identification of Ferroptosis-Related Differentially Expressed Genes

**Figure 2:**
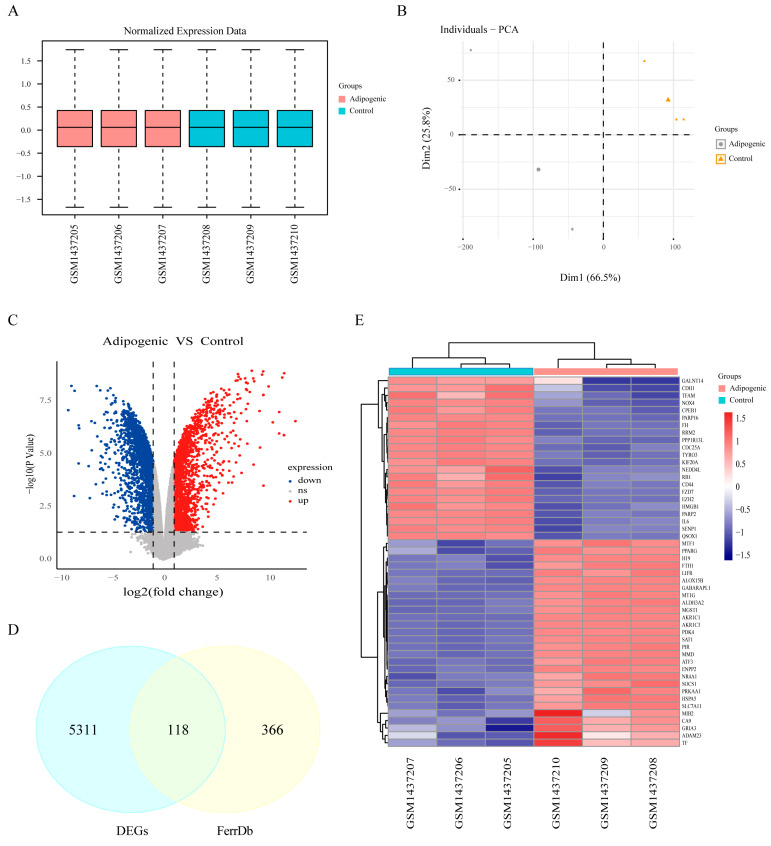
(**A**) Boxplot and (**B**) PCA results demonstrating that the data were well normalized. (**C**) Volcano plot of gene expression profiles according to data from the GSE59450 dataset. The blue dots indicate downregulated genes, whereas the red dots indicate upregulated genes. (**D**) Venn diagram illustrating ferroptosis-related differentially expressed genes. (**E**) Heatmap of the top 50 ferroptosis-related DEGs with the greatest fold changes in expression.

We subsequently downloaded the GSE59450 dataset, which contains data related to MSC adipogenic differentiation; the samples were categorized into adipogenic and control groups. After normalization, boxplots and PCA demonstrated that the gene expression data were well correlated and reliable (Figure 2A,B). We performed differential expression analysis between the adipogenic and control groups and identified a total of 5429 DEGs, including 2143 downregulated genes and 3286 upregulated genes (Figure 2C). These DEGs subsequently intersected with 484 ferroptosis-related genes, resulting in the identification of 118 ferroptosis-related DEGs, as illustrated in the Venn diagram (Figure 2D). The top 50 DEGs with the greatest fold changes were selected for heatmap visualization and comparison (Figure 2E).

### 3.3. Functional Enrichment Analysis

**Figure 3:**
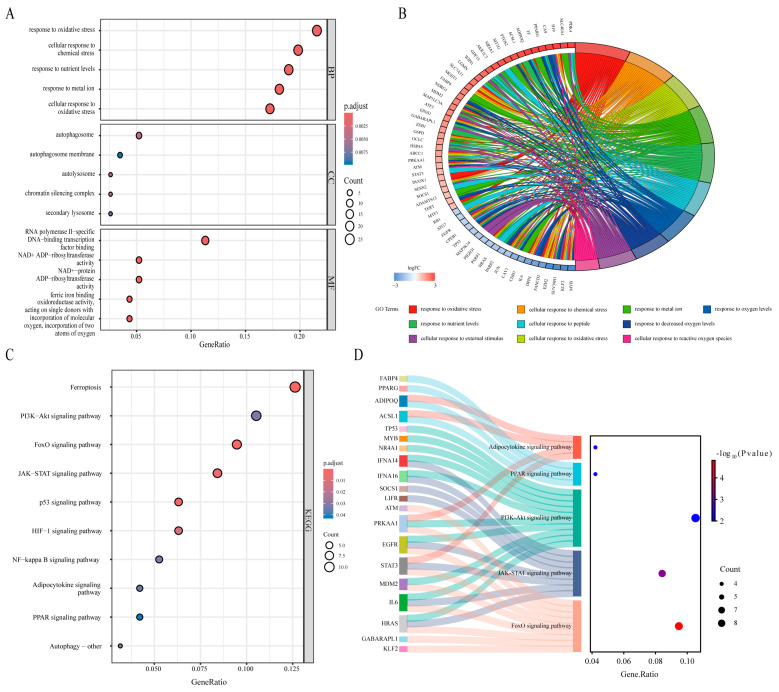

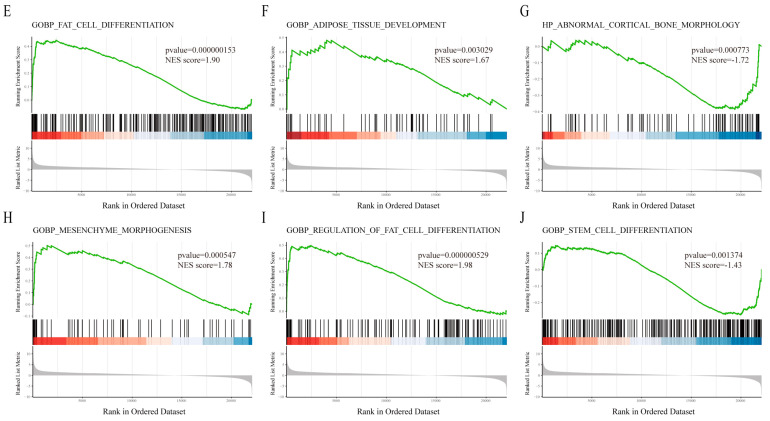
(**A**) GO enrichment analysis of FRDEGs. (**B**) Chord diagram illustrating the top 10 enriched biological processes. (**C**) KEGG pathway enrichment analysis of FRDEGs. (**D**) Sankey diagram visualizing the relationships between genes enriched in significant KEGG pathways. (**E**–**J**) GSEA of data obtained from the GSE59450 dataset.

To comprehensively assess the biological functions of FRDEGs, we first performed Gene Ontology (GO) and Kyoto Encyclopedia of Genes and Genomes (KEGG) enrichment analyses. GO functional analysis revealed that these genes are involved primarily in biological processes such as the response to oxidative stress and the cellular response to chemical stress. These genes are associated with cellular components such as the autophagosome membrane and secondary lysosomes, and they regulate molecular functions, including ferric iron binding, NAD+ ADP-ribosyltransferase activity, and NAD+-protein ADP-ribosyltransferase activity. The enrichment results are presented in a bubble plot (Figure 3A). The top 10 biological processes (BPs) were used to generate a chord diagram (Figure 3B).

KEGG pathway enrichment analysis revealed that the FRDEGs are involved in multiple signaling pathways related to MSC adipogenic differentiation, including the FoxO, PPAR, adipocytokine, JAK-STAT, and PI3K-Akt signaling pathways (Figure 3C). Additionally, we constructed a Sankey diagram to visualize the relationships between the enriched genes and key KEGG pathways (Figure 3D). Furthermore, gene set enrichment analysis (GSEA) revealed significant enrichment of the DEGs identified from the GSE59450 dataset in pathways related to fat cell differentiation, adipose tissue development, abnormal cortical bone morphology, mesenchyme morphogenesis, the regulation of fat cell differentiation, and stem cell differentiation (Figure 3E–J).

In conclusion, our findings indicate that the FRDEGs we identified are enriched in pathways associated with the regulation of MSCs adipogenic differentiation. These results suggest that FRDEGs play a crucial role in the adipogenic differentiation process of MSCs.

### 3.4. PPI Network Construction and Identification of Hub Genes

**Figure 4:**
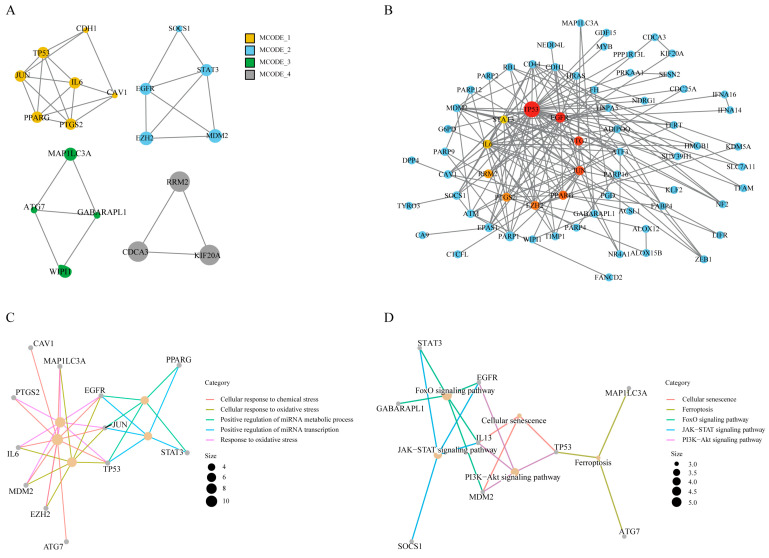
(**A**) The MCODE algorithm revealed four gene clusters. (**B**) The Betweenness (BC) algorithm of the CytoNCA plugin was used to construct a PPI network consisting of 118 genes, and the size and color depth of the circles indicate the score. The top 10 hub genes, identified by intersection using the MCODE algorithm, are marked in red. (**C**,**D**) GO enrichment analysis and KEGG pathway analysis of ferroptosis-related hub genes.

The 118 FRDEGs were imported into the STRING database, and Cytoscape was used to construct a PPI network of these genes. This network consisted of 115 nodes and 165 edges, with an average local clustering coefficient of 0.463, which indicated significant PPI enrichment (*p* < 0.01) (Supplementary Figure S1). The MCODE algorithm was employed to identify four highly connected modules, which included 19 genes (Figure 4A). Scores for each gene were subsequently calculated on the basis of the Betweenness (BC) algorithm of the CytoNCA plugin, and the top 10 hub genes were selected. Larger scores correspond to a greater circle size and color intensity. Ultimately, TP53, EGFR, ATG7, JUN, PPARG, EZH2, PTGS2, RRM2, IL6, and STAT3 were found to play crucial roles in the network according to both CytoHubba and MCODE (Figure 4B).

GO enrichment analysis of these 10 hub genes revealed that they were enriched in biological processes (BP) terms such as the cellular response to chemical stress, the response to oxidative stress, and the cellular response to oxidative stress (Figure 4C). KEGG pathway enrichment analysis revealed significant involvement of the genes in pathways such as the FoxO signaling pathway, JAK-STAT signaling pathway, cellular senescence pathway, and PI3K-Akt signaling pathway (Figure 4D). Consistent with previous findings, ferroptosis-related hub genes were significantly enriched in the oxidative stress and adipogenic differentiation pathways, suggesting that the oxidative stress induced by ferroptosis-related hub genes plays a critical role in MSC adipogenic differentiation.

### 3.5. Validation of the Diagnostic Value of Ferroptosis-Related Hub Genes

**Figure 5:**
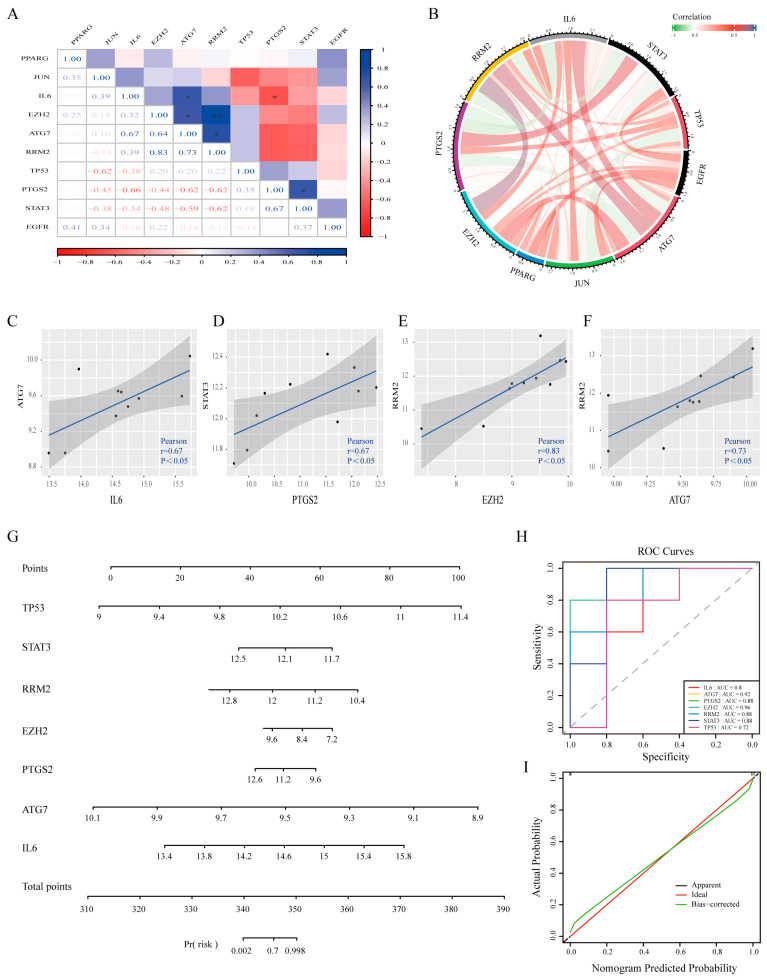
(**A**) Correlation heatmap of ferroptosis-associated hub genes. (**B**) Correlation chord diagram of ferroptosis-associated hub genes. (**C**–**F**) Scatter plots depicting the four most strongly correlated FRHG pairs. (**G**) Construction of the nomogram model based on the selected FRHGs. (**H**) ROC curve and the area under the curve (AUC). (**I**) Calibration curve illustrating the diagnostic ability of the nomogram model. * *p* < 0.05, ** *p* < 0.01.

To evaluate the functional importance and potential diagnostic value of ferroptosis-related hub genes (FRHGs), we first generated correlation heatmaps (Figure 5A) and correlation chord plots (Figure 5B) for the ferroptosis-related hub genes, and significant expression correlations were found among six gene pairs. Correlation scatter plots for the four FRHG pairs with the highest expression correlations were generated (Figure 5C–F). Significant expression correlations were identified between IL6 and ATG7 (Figure 5C, r = 0.67), PTGS2 and STAT3 (Figure 5D, r = 0.67), EZH2 and RRM2 (Figure 5E, r = 0.83), and ATG7 and RRM2 (Figure 5F, r = 0.73).

We subsequently performed ROC curve analysis of these 10 ferroptosis-related hub genes using the GSE35956 dataset, which contained data from osteoporosis patients, to assess their diagnostic value for osteoporosis. The results (Supplementary Figure S2) revealed the following AUC values for each gene: ATG7 (0.92), EGFR (0.64), EZH2 (0.96), IL6 (0.80), JUN (0.68), PPARG (0.52), PTGS2 (0.88), RRM2 (0.88), STAT3 (0.88), and TP53 (0.72). Thus, the results indicated that these hub genes have considerable potential for OP diagnosis.

Furthermore, we developed an OP risk prediction model, and to increase its predictive accuracy, we selected genes with AUC values greater than 0.7 for construction. This model is presented as a nomogram of all genes contributing to its construction in Figure 5G. The predictive efficacy of this model for OP was validated through ROC analysis (Figure 5H). Calibration curves further confirmed the reliability of the model (Figure 5I). Overall, this model demonstrates excellent diagnostic value for the early prediction of osteoporosis and assessment of osteoporosis risk.

### 3.6. Validation of the Expression of Ferroptosis-Related Hub Genes

**Figure 6:**
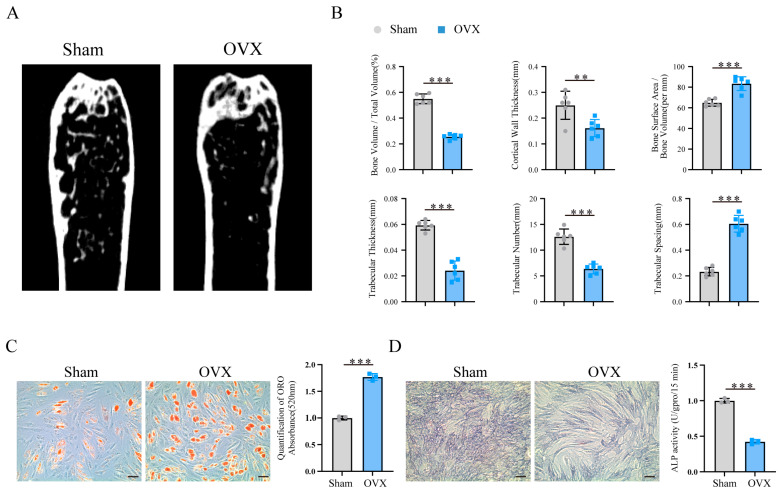

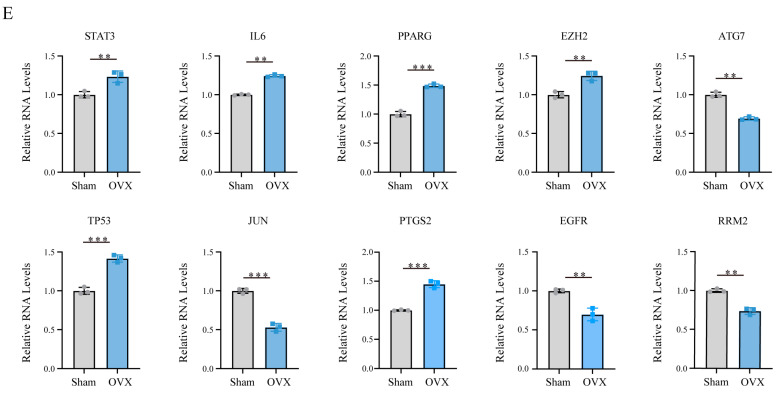
(**A**) Micro-CT imaging results. (**B**) Comparison of micro-CT bone microstructure parameters between the sham and OVX groups. (**C**,**D**) Oil Red O staining and alkaline phosphatase staining results for the Sham and OVX groups. The scale bar in the images represents 50 μm. (**E**) qRT-PCR analysis of the mRNA expression levels of 10 ferroptosis-related hub genes in the sham and OVX groups. All the data are presented as the means ± SDs, with n = 3 per group, ** *p* < 0.01, and *** *p* < 0.001. All experiments were independently repeated three times.

In osteoporosis patients, adipogenic differentiation is increased, whereas osteogenic differentiation is reduced [22,23]. To validate the expression of the 10 hub genes in osteoporosis, we constructed an ovariectomized (OVX) mouse model. Micro-CT analysis revealed that, compared with the sham group, the OVX group presented typical indications of bone loss, including significant decreases in the bone volume fraction (BV/TV), cortical wall thickness, trabecular thickness, and trabecular number, whereas the trabecular spacing and bone area/bone volume ratio (BA/BV) were increased (Figure 6A,B).

In addition, MSCs were isolated from the femurs of the mice and induced to undergo adipogenic and osteogenic differentiation. Oil Red O staining and ALP staining revealed that the MSCs from the OVX group presented increased adipogenic differentiation capacity and reduced osteogenic differentiation capacity (Figure 6C,D).

We subsequently performed qRT-PCR to assess the expression levels of these 10 hub genes. Compared with the sham group, the OVX group presented significant downregulation of ATG7, JUN, EGFR, and RRM2 and significant upregulation of TP53, PPARG, EZH2, IL6, PTGS2, and STAT3 (*p* < 0.05) (Figure 6E). These results suggest that our bioinformatics data mining approach was highly reliable.

### 3.7. Prediction of Drugs Affecting the Expression of Ferroptosis-Related Hub Genes

Drug intervention remains the primary approach in the treatment of osteoporosis. Target-drug interaction analysis can reveal interactions between drugs and targets that are critical for understanding the structural features necessary for receptor sensitivity [24]. This study identified 10 ferroptosis-related hub genes as potential drug targets, and the top 10 candidate drugs predicted to target these genes were selected from the DSigDB database on the basis of *p* values (Table 1). We subsequently retrieved the structural formulas of these drugs from the DrugBank database (Supplementary Figure S3).

Several of the compounds have been extensively studied, and their pharmacological effects have been confirmed. For example, gefitinib, doxorubicin, thalidomide, and staurosporine are anticancer drugs; 3-methyladenine is an autophagy inhibitor; piroxicam and sulindac sulfide (EINECS 250-892-2 CTD 00001193) are nonsteroidal anti-inflammatory compounds; and tyrphostin AG-1478 hydrochloride (170449-18-0 CTD 00003361) is a selective EGFR tyrosine kinase inhibitor with antiviral properties. However, their pharmacological effects are unrelated to osteoporosis treatment, and they are associated with known severe side effects, such as cardiotoxicity and myelosuppression [25,26].

Notably, we also identified pterostilbene and masoprocol, two natural compounds with significant antioxidant properties. As plant-derived compounds, they have a high safety profile [27,28]. Furthermore, the antioxidant effects of these compounds may help mitigate bone loss caused by oxidative stress, indicating their potential for osteoporosis prevention and treatment. Therefore, pterostilbene and masoprocol were selected as candidate compounds for further investigation.

### 3.8. Molecular Docking Analysis and Functional Validation of the Candidate Drugs

**Figure 7:**
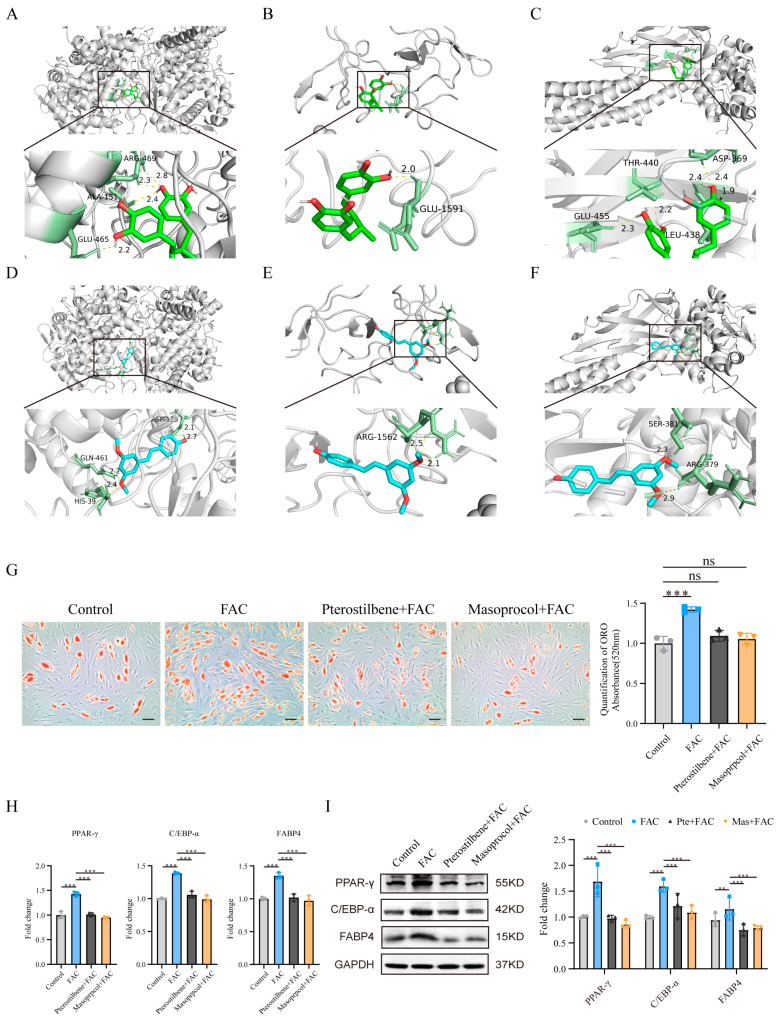
Molecular docking analysis and functional validation of the candidate drugs. (**A**) Molecular docking between masoprocol and PTGS2. (**B**) Molecular docking between masoprocol and TP53. (**C**) Molecular docking between masoprocol and STAT3. (**D**) Molecular docking between pterostilbene and PTGS2. (**E**) Molecular docking between pterostilbene and TP53. (**F**) Molecular docking between pterostilbene and STAT3. (**G**) ORO staining and quantitative analysis of MSC adipogenic differentiation. The scale bars in the images represent 50 μm. (**H**) qRT-PCR and (**I**) Western blotting analysis of the expression of the adipogenic markers PPAR-γ, C/EBP-α, and FABP4 in MSCs. All the data are presented as the means ± SDs, with n = 3 per group, ** *p* < 0.01, and *** *p* < 0.001. ns, no significant difference. All experiments were independently repeated three times.

To assess the affinity of drug candidates for their targets and elucidate the druggability of the drug targets, molecular docking analysis was performed in this study. AutoDock Vina v.1.2.2 was used to identify the binding sites and interactions between the candidate drugs and the proteins encoded by their target hub genes and to calculate the binding energies for each interaction. Each candidate drug bound to its protein target via visible hydrogen bonds and strong electrostatic interactions. In addition, the binding pocket of each target was successfully occupied by four drug candidates. The results indicated that the two candidate small molecules exhibited good binding affinity with three key ferroptosis-related genes products (Table 2). The structural information and molecular docking conformations of the small molecules are shown in Figure 7A–F.

We further validated the effects of the candidate drugs pterostilbene and masoprocol on MSC adipogenic differentiation. The Oil Red O staining results revealed that pterostilbene and masoprocol significantly inhibited FAC-induced MSC adipogenic differentiation (Figure 7G). qRT-PCR and Western blotting further revealed that FAC treatment significantly increased the expression of the adipogenesis-related markers PPAR-γ, C/EBP-α, and FABP4, whereas pterostilbene and masoprocol effectively reversed the FAC-induced upregulation of these markers (Figure 7H,I). These results suggest that pterostilbene and masoprocol can effectively alleviate the enhancement of MSC adipogenic differentiation induced by iron overload by inhibiting ferroptosis in MSCs.

## 4. Discussion

Osteoporosis is a systemic metabolic disease characterized by low bone mass and impaired bone microstructure, which leads to increased bone fragility and susceptibility to fractures [29]. The strict allocation of MSC lineages plays a crucial role in maintaining bone homeostasis, and their senescence or death is often considered one of the initiating factors in the pathogenesis of osteoporosis [30]. Ferroptosis is a newly discovered form of cell death and is considered an independent risk factor for osteoporosis. Numerous studies have shown that targeting ferroptosis can prevent the progression of osteoporosis [31], but whether ferroptosis can affect the balance of MSC osteoblast–adipocyte differentiation and the potential mechanism underlying this influence in osteoporosis remain unclear. Here, we first demonstrated that inhibiting ferroptosis can reduce the adipogenic differentiation of MSCs, and then we used bioinformatics techniques to identify key hub genes related to ferroptosis, including TP53, EGFR, ATG7, JUN, PPARG, EZH2, PTGS2, RRM2, IL6, and STAT3. We then confirmed through ROC analysis that these genes play important roles in the development of osteoporosis and may serve as potential therapeutic targets. Finally, we successfully identified two small-molecule compounds, pterostilbene and masoprocol, from the DrugBank database, validated their binding affinity to the protein products of hub genes through molecular docking technology, and confirmed that they can reduce MSC adipogenic differentiation by inhibiting ferroptosis.

To determine the role of ferroptosis-related differentially expressed hub genes in MSC adipogenic differentiation and the related mechanisms, we performed functional enrichment analysis. According to KEGG pathway analysis, the hub genes were enriched in pathways such as the FoxO signaling pathway, the JAK-STAT signaling pathway, cellular senescence, and the PI3K-Akt signaling pathway. According to GO enrichment analysis, the genes were enriched in the BP terms cellular response to chemical stress, response to oxidative stress, and cellular response to oxidative stress. Oxidative stress is the result of excessive accumulation of ROS within the cell; at low levels, ROS can act as second messengers to regulate various physiological cell responses [32]. Experiments by Chia-Hua Lin et al. [33] demonstrated that ROS, by inhibiting the expression of SIRT1, upregulated the expression of the adipogenic transcription factors KLF5, C/EBPβ, PPARγ, and LEPTIN while downregulating the expression of the osteogenic transcription factors RUNX2, c-MAF, and COL1A in MSCs, leading to increased MSC adipogenic differentiation and decreased osteogenic differentiation. In addition, Fatemeh Atashi et al. [34] detailed the effects of ROS on MSC adipogenic and osteogenic differentiation and reported that the induction of osteogenesis is greatest in the absence of ROS, whereas the induction of adipogenesis is greatest in the presence of ROS. As ferroptosis-related differentially expressed hub genes are enriched in pathways related to adipogenesis and oxidative stress, this result suggests a potential association between ferroptosis-induced oxidative stress and MSC adipogenic differentiation.

Next, we validated the clinical value of ferroptosis-related differentially expressed hub genes. The TP53 gene encodes the p53 transcription factor, one of the most common tumor suppressor genes. Evidence [35] has shown that p53 can reduce cystine uptake by inhibiting the expression of the key component SLC7A11 of the cystine/glutamate reverse transporter, thereby decreasing the cellular antioxidant capacity and exacerbating ferroptosis. Siddaraju V Boregowda et al. [36] demonstrated that the deletion of p53 in MSCs reduces mitochondrial ROS levels and inhibits the expression of PPARG, thereby preventing the initiation of adipogenesis. Moreover, in a study by Tao Yu et al. [37], both in vivo and in vitro experiments revealed that TP53 gene expression and serum p53 levels were increased in osteoporosis patients and in mouse models of osteoporosis. These elevated p53 levels were associated with reduced bone mass, and these changes were partially reversed by knocking down p53. These findings suggest that p53 may play a central role in the onset and progression of osteoporosis. In human mammary epithelial cells, activated EGFR induces ROS production through the MAPK signaling pathway, thereby promoting ferroptosis [38]. However, in osteoblasts, EGFR activates the MAPK/ERK pathway, stimulating the expression of the downstream EGR transcription factor 2, thereby promoting cell growth and proliferation and stimulating bone formation [39]. Furthermore, Guanqiao Liu et al. [40] reported that the phosphorylation level of EGFR on the bone endosteal surface in 15-month-old middle-aged mice was significantly lower than that in 3-month-old young mice. Inhibition of the EGFR signaling pathway through the ERK1/2 pathway promoted osteoprogenitor cell senescence and resulted in decreased bone formation on the cortical bone endosteal surface. EZH2 is a histone methyltransferase that trimethylates histone H3 lysine 27 (H3K27). EZH2 is highly expressed in hepatocellular carcinoma (HCC) and can regulate the levels of histone H3K27me3 in the promoter region of TFR2, maintaining iron homeostasis in HCC and inhibiting ferroptosis [41]. However, Huan Jing et al. [42] reported that in osteoporosis, EZH2 silences the Wnt/β-catenin signaling pathway in MSCs by directly increasing the levels of H3K27me3 at the Wnt protein promoter, inducing MSC differentiation into adipocytes. Using lentivirus-expressed shRNA to knock down EZH2 effectively slowed the progression of osteoporosis. ATG7 is a key effector enzyme in the autophagy process and plays a central role in the initiation of autophagy. Autophagy-driven ferritin degradation has been shown to lead to iron accumulation, triggering oxidative stress via the Fenton reaction in the cell and inducing ferroptosis. In addition, ATG7 plays a crucial role in regulating oxidative stress and its induced cell death response. Li et al. [43] reported that ATG7 conditional knockout (cKO) mice exhibit decreased bone mass both during development and in adulthood and that the absence of ATG7 in osteoblasts leads to endoplasmic reticulum stress, which in turn inhibits osteoblast mineralization and promotes cell apoptosis. RRM2 is a ribonucleotide reductase subunit responsible for catalyzing the synthesis of deoxyribonucleotides. Many studies have shown that RRM2 is an important regulator of ferroptosis [44]. Yang Y et al. [45] reported that RRM2 exerts an antiferroptotic effect on liver cancer cells by maintaining glutathione synthesis. In mouse embryonic fibroblasts (MEFs), RRM2 overexpression promotes osteogenic differentiation, whereas RRM2 knockdown inhibits this process. RRM2 may regulate the osteogenic differentiation of MEFs via the classical Wnt/β-catenin signaling pathway, providing a potential target for the treatment of osteoporosis [46].

Additionally, PPARG, PTGS2, IL6, STAT3, and JUN have been reported to play important roles in the development of osteoporosis. For example, PPARG is a biomarker for osteoporosis, and the PPAR-γ protein it encodes is a key regulator of adipocyte differentiation. STAT3 is a downstream effector molecule of the IL6 signaling pathway, and IL6 promotes osteoclast formation and bone resorption by activating the JAK-STAT3 signaling pathway [47]. The transcription factor JUN is considered a new therapeutic target for osteoporosis-related fractures. JUN can induce the hedgehog signaling pathway to expand the osteoprogenitor cell population and promote their differentiation towards osteoblasts. In a hole defect model, JUN accelerated bone growth and healing [48]. Furthermore, PTGS2 is considered a marker of ferroptosis. Shuangliu Chen et al. [49] reported that curcumin inhibits MSC ferroptosis induced by iron overload through its action on PTGS2, thereby preventing the impairment of MSC osteogenic differentiation and mineralization. In summary, most of these genes have been shown to affect the progression of osteoporosis. Our ROC analysis results also revealed that these 10 key genes have high AUC values, making them potential biomarkers for the diagnosis of osteoporosis due to its association with ferroptosis. Moreover, subsequent qPCR experiments confirmed that these genes were differentially expressed in the osteoporosis group. Overall, our results provide new directions for selecting biomarkers and exploring therapeutic targets for osteoporosis.

Drugs are the main treatment for osteoporosis. Using molecular docking technology, we successfully identified two small-molecule compounds from the DrugBank database. Pterostilbene (PTE) is a resveratrol analogue found in blueberries that has antioxidant, antitumor, and anti-inflammatory activities. It inhibits cellular inflammation, oxidative stress, apoptosis, and ferroptosis [50]. Clinical research by Yu et al. [51] revealed that PTE treatment in rats subjected to 30 min of myocardial ischemia followed by 3 h of reperfusion significantly improved cardiac function, reduced myocardial infarction, and alleviated myocardial apoptosis after I/R injury. In a type 2 DM rat model, PTE was shown to potentially regulate liver metabolic processes, including glycolysis and gluconeogenesis, thereby increasing the glucose concentration and promoting glucose homeostasis [52]. Furthermore, Lee et al. [53] confirmed that PTE can ameliorate OA-related damage and promote ECM (extracellular matrix) repair, suggesting that PTE might have therapeutic potential in cartilage repair. Therefore, PTE is a bioactive phytochemical with significant medicinal potential and promising applications. Masoprocol is a lipoxygenase inhibitor isolated from the creosote bush that has been shown to decrease lipolytic activity in adipose tissue both in vivo and in vitro. Research by Jian Luo et al. [54] showed that the oral administration of masoprocol reduces blood glucose and triglyceride (TG) levels in type 2 diabetic rodent models. Additionally, Maya S. Gowri et al. [55] reported that oral gavage with masoprocol effectively reduced systolic blood pressure and plasma concentrations of insulin, free fatty acids, and triglycerides in fructose-induced hypertension model rats. In our study, we found that both pterostilbene and masoprocol can reduce MSC adipogenic differentiation by inhibiting ferroptosis, suggesting that these two small-molecule compounds could serve as novel agents for treating osteoporosis.

Overall, the innovation of this study lies in the finding that inhibiting ferroptosis can reduce MSC adipogenic differentiation in vitro. This study combines bioinformatics analysis with molecular docking techniques to identify ferroptosis-related OP genes and potential therapeutic small molecules associated with targeting ferroptosis. These findings suggest that oxidative stress induced by ferroptosis, which affects MSC adipogenic differentiation, may underlie the progression of osteoporosis. To date, research on the link between osteoporosis and ferroptosis is still limited, and this study fills a gap in this field. However, this study also has certain limitations. For example, although the ovariectomized mouse model effectively simulates the clinical features of postmenopausal osteoporosis and was used to verify the expression levels of key ferroptosis-related genes, further molecular and cellular experiments are needed to confirm whether these 10 key ferroptosis-related DEGs can serve as potential biomarkers for osteoporosis. Furthermore, the drugs that target these genes still require further research and clinical trials. In this context, our research findings are only predictive and require more experimental validation.

## Figures and Tables

**Table 1:** The top 10 recommended drugs.

Drug Names	*p*-Value	Adjusted *p*-Value	Genes
Gefitinib CTD 00003879	1.65 × 10^−8^	1.24 × 10^−5^	*STAT3*; *PTGS2*; *ATG7*; *TP53*
Doxorubicin CTD 00005874	1.85 × 10^−8^	1.24 × 10^−5^	*RRM2*; *STAT3*; *PTGS2*; *ATG7*; *TP53*; *EZH2*
Thalidomide CTD 00006858	8.15 × 10^−8^	3.65 × 10^−5^	*RRM2*; *STAT3*; *PTGS2*; *TP53*
Staurosporine CTD 00007273	1.73 × 10^−7^	5.80 × 10^−5^	*STAT3*; *PTGS2*; *ATG7*; *TP53*
Piroxicam CTD 00006571	3.07 × 10^−7^	8.25 × 10^−5^	*RRM2*; *STAT3*; *PTGS2*; *TP53*; *EZH2*
EINECS 250-892-2 CTD 00001193	5.13 × 10^−7^	1.15 × 10^−4^	*RRM2*; *STAT3*; *PTGS2*; *TP53*
Pterostilbene CTD 00003490	8.46 × 10^−7^	1.42 × 10^−4^	*STAT3*; *PTGS2*; *TP53*
Masoprocol CTD 00006416	1.19 × 10^−6^	1.78 × 10^−4^	*STAT3*; *PTGS2*; *TP53*
3-Methyladenine BOSS	8.46 × 10^−7^	1.42 × 10^−4^	*STAT3*; *ATG7*; *TP53*
170449-18-0 CTD 00003361	1.42 × 10^−6^	1.91 × 10^−4^	*STAT3*; *PTGS2*; *TP53*

**Table 2:** Molecular docking scores of masoprocol and pterostilbene with the protein products of three key ferroptosis genes (kcal/mol).

Compound	PTGS2	TP53	STAT3
Masoprocol	−8.8	−8.3	−6.6
Pterostilbene	−8.1	−7.4	−6.2

## Data Availability

All data generated or analyzed in this study are included in the article and its supplementary files.

## Supplementary Materials

The following supporting information can be downloaded at https://www.mdpi.com/article/10.3390/biomedicines13040940/s1. Figure S1: PPI network diagram showing 115 nodes and 165 edges; Figure S2: ROC curve analysis for the 10 Hub genes; Figure S3: Chemical structures of the 10 predicted drugs; Figure S4: Original gels images of western blots in Figure 1E and Figure 7I; Table S1: Primer sequences for real-time PCR.
