# Supplementary material for: Bioinformatics-Based Analysis of Ferroptosis-Related Biomarkers and the Prediction of Drugs Affecting the Adipogenic Differentiation of MSCs
Source: Biomedicines. 2025 Apr 11;13(4):940. doi: 10.3390/biomedicines13040940 (PMC12025237; doi:10.3390/biomedicines13040940)
Supplement: Supplementary file 1 [file biomedicines-13-00940-s001.zip › Supplementary File S1--Table S1.pdf]

Supplementary Table S1. Primer sequences for real-time PCR

Primer sequence		
TP53	Forward	5'-CAGCACATGACGGAGGTTGT-3'
	Reverse	5'-TCATCCAAATACTCCACACGC-3'
EGFR	Forward	5'-TTGCCGCAAAGTGTGTAACG-3'
	Reverse	5'-GTCACCCCTAAATGCCACCG-3'
ATG7	Forward	5'-ATGATCCCTGTAACTTAGCCCA-3'
	Reverse	5'-CACGGAAGCAAACAACTTCAAC-3'
JUN	Forward	5'-TCCAAGTGCCGAAAAAGGAAG-3'
	Reverse	5'-CGAGTTCTGAGCTTTCAAGGT-3'
PPARG	Forward	5'-GGGATCAGCTCCGTGGATCT-3'
	Reverse	5'-TGCACTTTGGTACTCTTGAAGTT-3'
EZH2	Forward	5'-AATCAGAGTACATGCGACTGAGA-3'
	Reverse	5'-GCTGTATCCTTCGCTGTTTCC-3'
PTGS2	Forward	5'-TAAGTGCGATTGTACCCGGAC-3'
	Reverse	5'-TTTGTAGCCATAGTCAGCATTGT-3'
RRM2	Forward	5'-GTGGAGCGATTTAGCCAAGAA-3'
	Reverse	5'-CACAAGGCATCGTTTCAATGG-3'
IL6	Forward	5'-ACTCACCTCTTCAGAACGAATTG-3'
	Reverse	5'-CCATCTTTGGAAGGTTCAGGTTG-3'
STAT3	Forward	5'-CAGCAGCTTGACACACGGTA-3'
	Reverse	5'-AAACACCAAAGTGGCATGTGA-3'
PPAR-γ	Forward	5'-GGGATCAGCTCCGTGGATCT-3'
	Reverse	5'-TGCACTTTGGTACTCTTGAAGTT-3'
C/EBPα	Forward	5'-TATAGGCTGGGCTTCCCCTT-3'
	Reverse	5'-AgCTTTCTGGTGTGACTCGG-3'
FABP4	Forward	5'-ACTGGGCCAGGAATTTGACG-3'
	Reverse	5'-CTCGTGGAAGTGACGCCTT-3'
GAPDH	Forward	5'-GGAGCGAGATCCCTCCAAAAT-3'
	Reverse	5'-GGCTGTTGTCATACTTCTCATGG-3'

Abbreviations: *TP53*, Tumor Protein P53; *EGFR*, Epidermal Growth Factor Receptor; *PPAR-γ*, peroxisome proliferator-activated receptor gamma; *C/EBP-α*, CCAAT/enhancer binding protein alpha; *FABP4*, fatty acid binding protein 4; *ATG7*, Autophagy Related 7; *JUN*, Jun Proto-Oncogene, AP-1 Transcription Factor Subunit; *PPARG*, Peroxisome Proliferator Activated Receptor Gamma ; *EZH2*, Enhancer Of Zeste 2 Polycomb Repressive Complex 2 Subunit; *PTGS2*, Prostaglandin-Endoperoxide Synthase 2; *RRM2*, Ribonucleotide Reductase Regulatory Subunit M2; *IL6*, Interleukin 6; *STAT3*, Signal Transducer And Activator Of Transcription 3; *GAPDH*, glyceraldehyde-3-phosphate dehydrogenase.
