# Supplementary material for: Bioinformatics-Based Analysis of Ferroptosis-Related Biomarkers and the Prediction of Drugs Affecting the Adipogenic Differentiation of MSCs
Source: Biomedicines. 2025 Apr 11;13(4):940. doi: 10.3390/biomedicines13040940 (PMC12025237; doi:10.3390/biomedicines13040940)
Supplement: Supplementary file 1 [file biomedicines-13-00940-s001.zip › Supplementary File S2--Figures S1-S3.pdf]

Supplementary Figure S1 PPI network diagram showing 115 nodes and 165 edges.

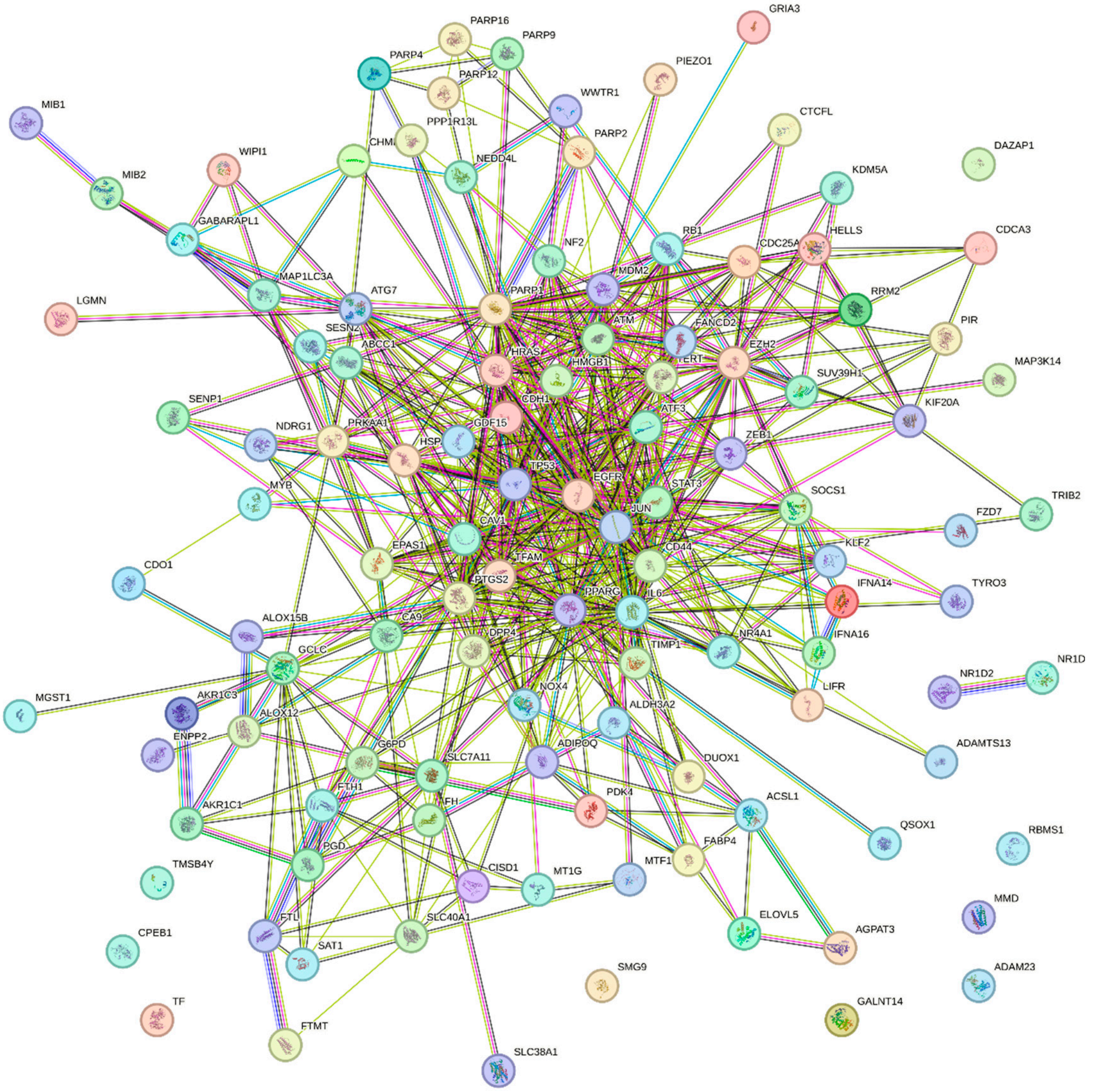

Supplementary Figure S2 ROC curve analysis for the 10 Hub genes.

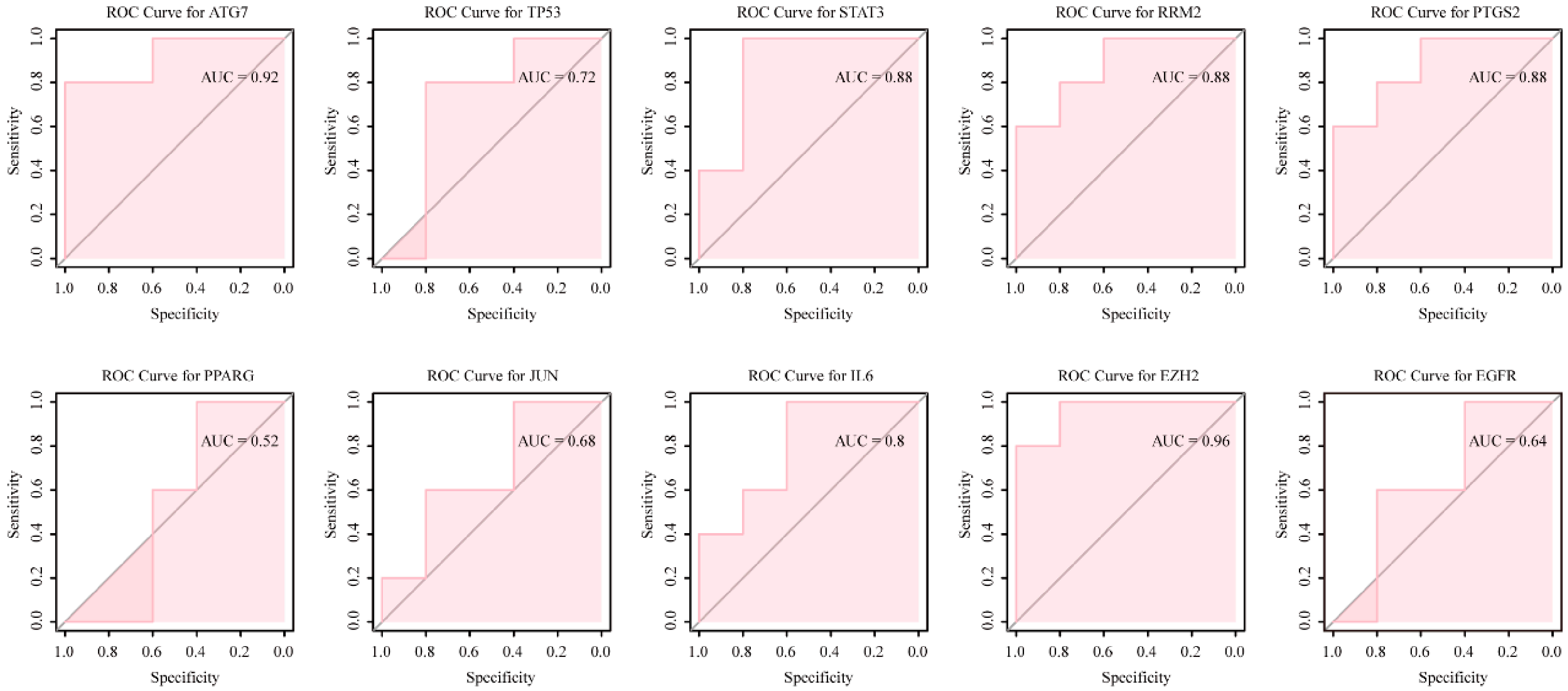

Supplementary Figure S3 Chemical structures of the 10 predicted drugs.

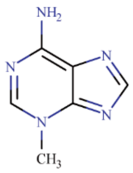

3-Methyladenine

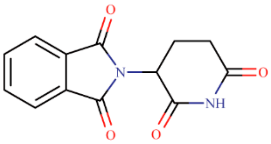

Thalidomide

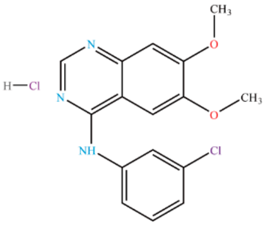

170449-18-0 CTD 00003361

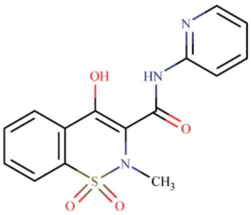

Piroxicam

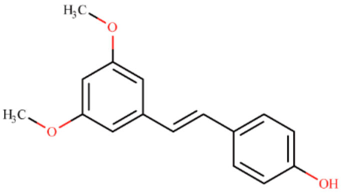

Pterostilbene

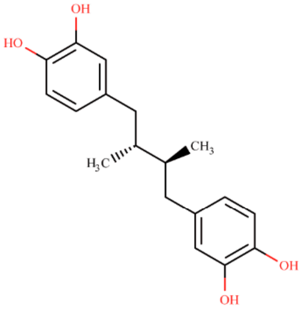

Masoprocol

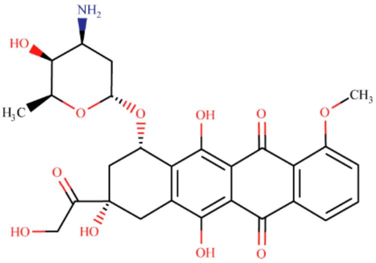

Doxorubicin

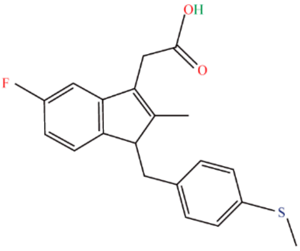

EINECS 250-892-2

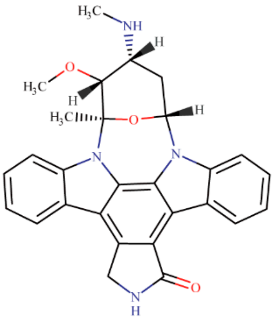

Staurosporine

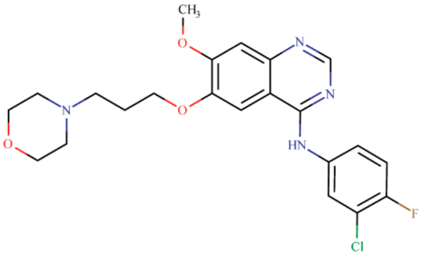

Gefitinib
