# Supplementary figures and images for: Bioinformatics-Based Analysis of Ferroptosis-Related Biomarkers and the Prediction of Drugs Affecting the Adipogenic Differentiation of MSCs
Source: Biomedicines. 2025 Apr 11;13(4):940. doi: 10.3390/biomedicines13040940 (PMC12025237; doi:10.3390/biomedicines13040940)

Supplementary Figure S4

Fig.1E (in paper)

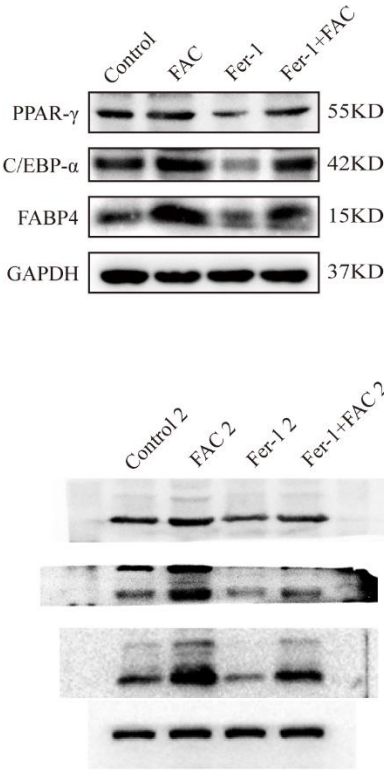

Raw data

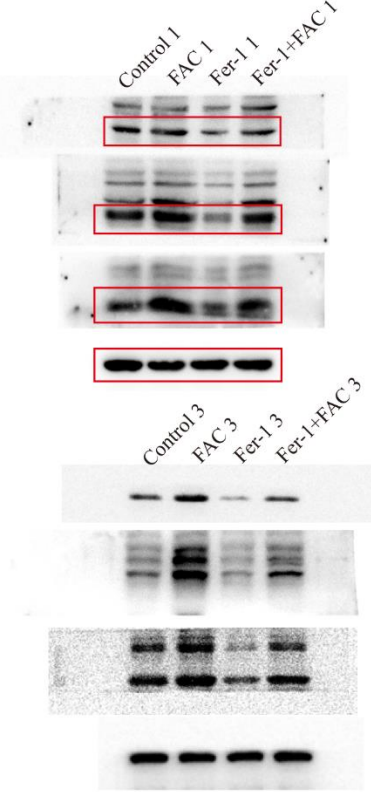

Fig.7I (in paper)

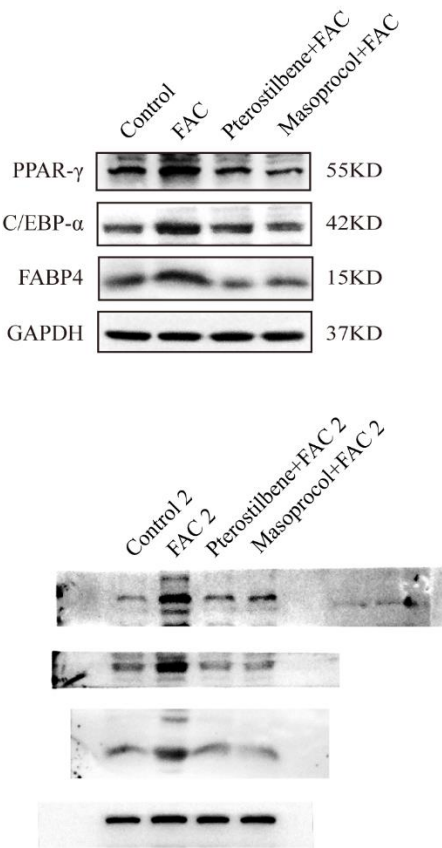

Raw data

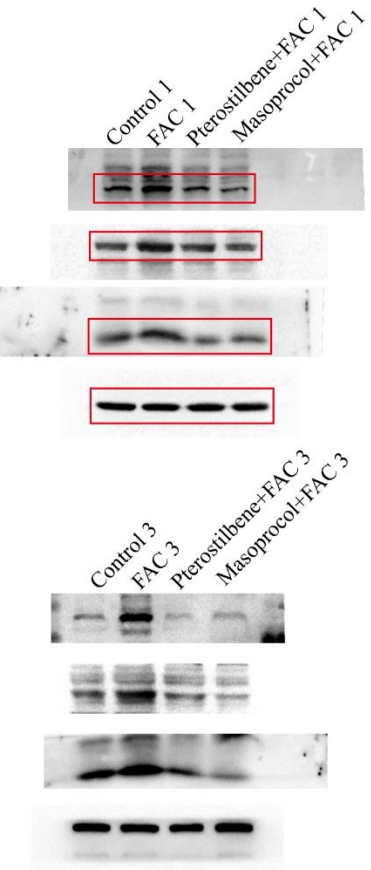

Supplement: Supplementary file 1 [file biomedicines-13-00940-s001.zip › Supplementary File S3--Figure S4.pdf]
